# Human Milk Banks in Iran: A Model for Integrating Global Best Practices within a Cultural and Religious Context

**DOI:** 10.34172/aim.35281

**Published:** 2026-01-01

**Authors:** Ahmadreza Afshar, Mohammadbagher Husseini, Shirin Khatibshahidi

**Affiliations:** ^1^Vice Chancellor of Clinical Affairs, Department of Orthopedics, Urmia University of Medical Sciences, Urmia, Iran; ^2^Pediatric Health Research Center, Tabriz University of Medical Sciences, Tabriz, Iran; ^3^Supervisor of Human Milk Bank, Tabriz University of Medical Sciences, Tabriz, Iran

## Dear Editor,

**Figure 1:**
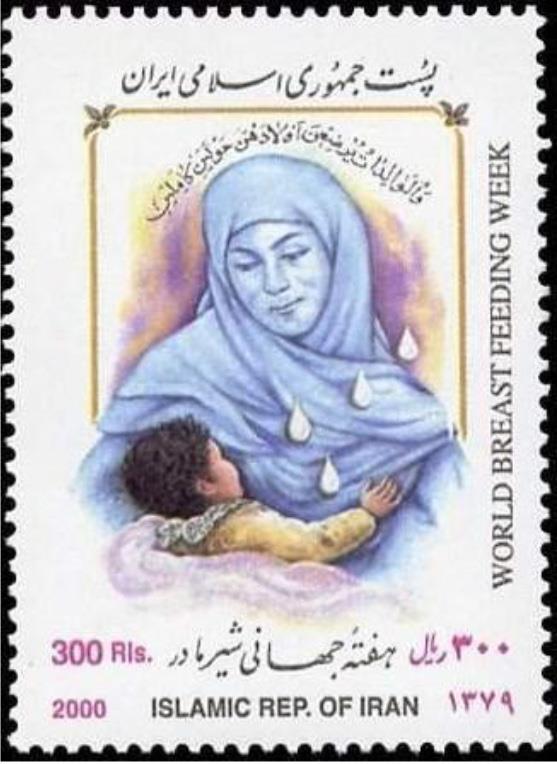
Postage stamp issued by Iran in 2019 to commemorate Mother Breastfeeding Week and promote the national breastfeeding policy

 The World Health Organization (WHO) and the United Nations International Children’s Emergency Fund (UNICEF) recommend infants to be exclusively breastfed by their biological mothers for the first six months of life to ensure optimal growth, development, and health. Accordingly, breastfeeding by the biological mother has been established as a governmental health policy worldwide, including in Iran. Each year, August 16–21 is recognized globally as Mother Breastfeeding Week. In 2019, Iran commemorated this occasion by issuing a postage stamp promoting and popularizing its breastfeeding policy (Figure 1).

 However, some mothers are unable to breastfeed or produce sufficient milk for their infants, necessitating alternative feeding options such as wet nursing or commercial formula. Milk shared through informal wet nursing is unpasteurized and may transmit infectious agents from donor to infant. Studies have shown that the intestinal flora of breastfed infants differ significantly from those fed through other means. Pasteurized donor human milk, endorsed by the WHO and UNICEF, has been established as the safest and most effective substitute for breast milk when a mother’s own milk is unavailable, particularly for preterm and low-birth-weight infants or those with metabolic and gastrointestinal disorders.^1-6^

 Human milk feeding reduces the length of hospital stays and the need for neonatal intensive care unit (NICU) admission. It also lowers the risk of retinopathy of prematurity, gastrointestinal infections, and necrotizing enterocolitis, thereby reducing neonatal mortality.^2-4^ Consequently, the global demand for human milk banks has increased steadily as part of efforts to improve newborn health outcomes.

 A human milk bank (HMB) is a nonprofit institution that selects, collects, screens, pasteurizes, stores, and distributes donated human milk to meet the nutritional needs of vulnerable newborns.^1,5,7^ The world’s first HMB was established in Vienna, Austria, in 1909.^1,7^ Since then, the number of milk banks has grown globally. As of September 2025, approximately 756 active HMBs exist worldwide, though their activities vary depending on cultural, religious, and operational standards.^5^

 In Iran, the establishment of HMBs was made possible through collaboration among the Ministry of Health and Medical Education (MOHME), charitable NGOs, and philanthropists, under the coordination of the Human Milk Bank Association of the Islamic Republic of Iran (HMBAI). In 2016, the first HMB in Iran and the Middle East was inaugurated at Al-Zahra Hospital, Tabriz University of Medical Sciences. Since then, Iran’s national HMB network has expanded rapidly. By October 2025, 16 active milk banks had been established in university-affiliated hospitals across the country, positioning Iran as the regional leader in HMB development (Table 1). The milk produced in these facilities serves both inpatients and outpatients and may be distributed to other hospitals as needed. Further expansion and interconnection of HMBs across the country are planned. The global HMB map available on Google confirms the presence of these centers in Iran.^1^

**Table 1:** Locations of established human milk banks in Iran (as of October 2025)

**University**	**Hospital**	**Established Year**
Tabriz University of Medical Sciences	Al-Zahra	2016
Ahvaz University of Medical Sciences	Emam Khomeini	2018
Shiraz University of Medical Sciences	Hazrat Zeynab	2019
Yazd University of Medical Sciences	Shahid Sadugi	2019
Mashhad University of Medical Sciences	Omolbanin	2019
Iran University of Medical Sciences	Akbarabadi	2019
Tehran University of Medical Sciences	Valiasr	2019
Kermanshah University of Medical Sciences	Emam Reza	2019
Kerman University of Medical Sciences	Afzalipour	2019
Isfahan University of Medical Sciences	Al-zahra	2021
Zahedan University of Medical Sciences	Ali-ebn-abitaleb	2022
Neyshabur University of Medical Sciences	Hakim	2024
Shahid Beheshty University of Medical Sciences	Mofid	2024
Alborz University of Medical Sciences	Kamaly	2024
Urmia University of Medical Sciences	Kosar	2025
Qom University of Medical Sciences	Khayerin	2025

 The success of Iran’s HMB network is reflected in the growing volume of collected milk and the increasing number of beneficiaries. According to the Neonatal Health Office of MOHME, the total volume of donated milk rose from 3,164 liters in 2021 to 5,498 liters in 2025. During the same period, the number of infants benefiting from HMB services increased from 2,951 to 5,523. These data illustrate the life-saving impact of HMBs in supporting at-risk newborns and align with Iran’s declining neonatal mortality rate.

 Beyond clinical care, HMBs in Iran function as centers for education and research. Educational activities target both healthcare providers and the general public to promote awareness of the benefits of breast milk donation. A PubMed search in October 2025 using the keywords *“Human Milk Bank”* and *“Iran”* yielded 12 scientific publications, underscoring the growing academic contribution of Iran’s HMB network.

 As with organ and tissue transplantation or blood donation, the evolution from traditional wet nursing to structured milk banking requires interdisciplinary collaboration and societal acceptance. A key success factor in Iran has been the consensus achieved among religious authorities regarding milk kinship—a concept central to Islamic law. Ethical frameworks ensuring donor–recipient anonymity have enabled the program’s full religious and cultural endorsement.

 The rapid, evidence-based, and culturally sensitive development of Iran’s human milk bank network illustrates how global health practices can be effectively localized. This experience provides a valuable model for other nations seeking to integrate human milk banking within diverse cultural and religious environments.
